# The Role of Nutrition and Nutritional Supplements in the Prevention and Treatment of Malnutrition in Chronic Obstructive Pulmonary Disease: Current Approaches in Nutrition Therapy

**DOI:** 10.1007/s13668-025-00613-8

**Published:** 2025-01-25

**Authors:** Tuğba Tuna, Gülhan Samur

**Affiliations:** 1 Faculty of Health Sciences, Department of Nutrition and Dietetics, Mudanya University, Bursa, Turkey; 2 Faculty of Health Sciences, Department of Nutrition and Dietetics, Hacettepe University, Ankara, Turkey

**Keywords:** COPD, Malnutrition, Nutritional therapy, Sarcopenia, Nutritional supplements

## Abstract

**Purpose of Review:**

Malnutrition is a significant comorbidity in Chronic Obstructive Pulmonary Disease (COPD), contributing to disease progression and reduced quality of life. This narrative review examines the role of nutritional therapy in the prevention and management of malnutrition in COPD, emphasizing evidence-based approaches and their clinical implications.

**Recent Findings:**

COPD patients face increased metabolic demands, systemic inflammation, and reduced dietary intake, resulting in muscle wasting, sarcopenia, and cachexia. Recent evidence highlights the efficacy of targeted nutritional strategies, including essential amino acid supplementation, omega-3 fatty acids, vitamin D, and antioxidants, in improving respiratory function, muscle strength, and patient well-being. Comprehensive nutritional assessments and personalized interventions are increasingly recognized as critical components of COPD care. However, long-term efficacy data remain limited.

**Summary:**

Nutritional therapy plays a pivotal role in managing malnutrition and improving clinical outcomes in COPD. This review synthesizes the latest evidence, identifies gaps in current research, and proposes strategies for integrating personalized nutrition into COPD care. Future studies are needed to establish the long-term benefits of these interventions and to develop tailored nutritional guidelines for COPD patients.

## Introduction

Chronic Obstructive Pulmonary Disease (COPD) is defined as “a common, preventable, and treatable condition characterized by persistent respiratory symptoms and irreversible airflow limitation due to airway and/or alveolar abnormalities, usually caused by significant exposure to harmful gases and particles” [1, 2]. With high morbidity and mortality rates, COPD has emerged as a significant global public health issue, imposing a growing social and economic burden [3]. Currently the fourth leading cause of death worldwide, COPD is projected to become the third leading cause by 2030, accounting for 8.6% of global deaths [4–6]. The disease primarily affects smokers or former smokers, with prevalence increasing with age [7]. Clinical symptoms such as shortness of breath, chronic coughing, excessive sputum production, and reduced exercise tolerance are hallmarks of COPD [8–10].

Malnutrition is a common and critical comorbidity in COPD, affecting nearly half of the patients due to unintentional weight loss and reduced appetite. Factors such as increased ventilatory demand, systemic inflammation, and physical inactivity exacerbate this condition [11]. Malnutrition significantly worsens disease progression, leading to more frequent exacerbations, social isolation, and increased mortality risk [4, 12]. Recognizing and addressing nutritional disorders early, alongside accompanying comorbidities, are vital elements of a holistic COPD management strategy. Nutritional interventions not only enhance disease prognosis but also improve quality of life for patients [13, 14, 15].

The objective of this review is to explore the role of nutrition in the prevention and treatment of malnutrition in COPD, with a focus on current nutritional therapy and nutritioanal supplements approaches. By synthesizing recent evidence, this review aims to highlight the critical interplay between nutrition and COPD management while identifying gaps in the current understanding of effective interventions.

## Malnutrition in COPD

Unintentional weight loss and malnutrition are common issues in COPD patients [16]. National and international clinical nutrition associations recommend regular screening to detect nutritional risk. Although different tools have been developed for these screenings, nutritional risk screening is essential in COPD patients [17]. About 1 in 3 hospitalized COPD patients and 1 in 5 outpatients are at risk of malnutrition. Malnutrition can develop gradually over several years or following exacerbations [18]. This condition is associated with lower quality of life, increased healthcare utilization, and higher healthcare costs [19–21].

**Fig. 1:**
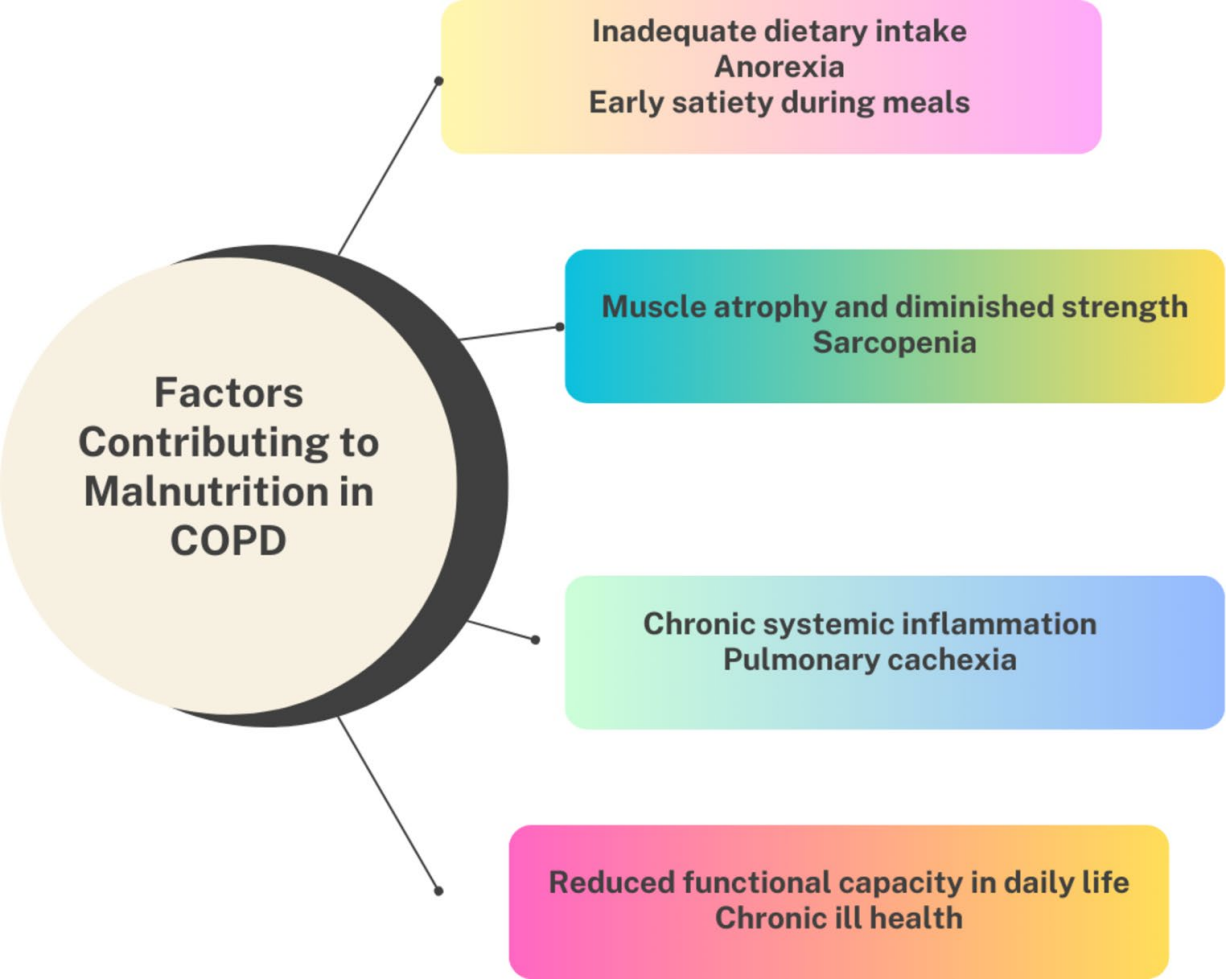
Etiology of malnutrition in COPD [22, 23]

The origin of malnutrition in COPD is complex and multifactorial (Fig. 1). In addition to disease symptoms, metabolic and pathophysiological factors, such as age-related factors and dietary habits, play an important role. Social factors can also contribute to inadequate nutrition. Malnutrition can adversely affect a patient’s overall health and physical-psychological well-being, often leading to exacerbations and hospitalizations. Additionally, weight loss is common due to increased difficulty in breathing, which can result in further physiological effects as the disease progresses [22, 23].

Sarcopenia is a syndrome characterized by decreased muscle mass, strength, and conditioning. Among the multiple complications of COPD, the prevalence of sarcopenia is 21.6%, varying based on age, disease severity, BMI, obstruction, dyspnea, and exercise capacity [24]. In COPD patients, atrophy is predominantly observed in the legs, especially in the thigh muscles. In severe COPD, unlike the normal aging process, muscle fibers shift from type I to type IIx [25]. Type II muscle fibers are particularly known to be more vulnerable to inflammation and hypoxia, leading to reduced muscle quality and decreased exercise tolerance. Hypoxia and hypercapnia negatively affect muscle function in COPD patients. Chronic hypoxemia and anemia lead to muscle hypoxia, resulting in reduced muscle mass, while hypercapnia contributes to muscle dysfunction and fiber atrophy. Aging is associated with changes in body composition, leading to a decrease in muscle mass and exercise tolerance, and may also lead to other health issues such as osteoporosis. Comorbidities are a primary cause of muscle loss in COPD patients. For example, chronic heart failure and chronic kidney disease play a significant role in the muscle wasting syndrome. Low-grade systemic inflammation leads to muscle atrophy and functional impairment [26].

Cachexia is also highly prevalent among COPD patients. It is a multifactorial metabolic syndrome characterized by fat loss and unintentional, progressive skeletal muscle wasting [27]. As the disease progresses, there is a gradual increase in the loss of muscle and fat mass in individuals. In this progressive process, multiple exacerbations contribute to disease progression and depletion of fat reserves [28]. Sarcopenia is an important phenotype in assessing nutritional status in COPD [29]. It is characterized by reduced skeletal muscle mass accompanied by decreased muscle strength. The coexistence of malnutrition and sarcopenia has a stronger impact on mortality than either condition alone. Osteoporosis often develops in COPD patients due to anorexia. The prevalence of this condition varies depending on diagnostic methods and disease severity. Vitamin D plays an essential role in regulating calcium metabolism, and low serum levels in COPD patients are associated with reduced bone mineral density. Considering the positive role of exercise in preventing osteoporosis, reduced exercise tolerance in COPD patients is recognized as a significant predictor of this condition [26, 30, 31].

### Consequences of Malnutrition in COPD

**Fig. 2:**
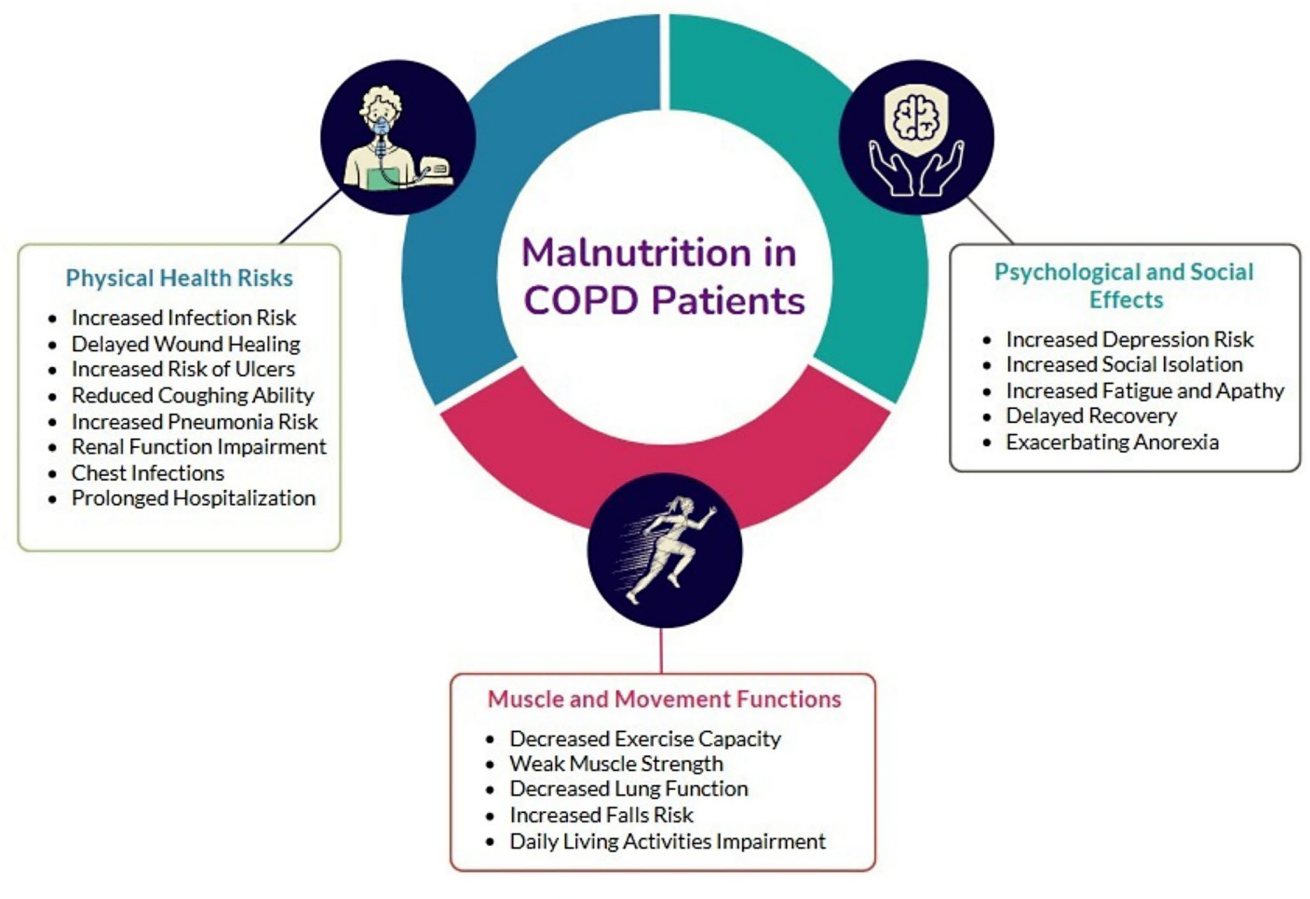
Consequences of malnutrition in COPD

Malnutrition, especially in patients with conditions like COPD, is associated with a wide range of severe and potentially life-threatening conditions and complications [32] (Fig. 2). This condition can lead to frequent exacerbations, hospitalizations, and a general deterioration in physiological status. Advancing age and worsening disease progression often intensify this effect and may result in multifaceted physiological impacts. This situation significantly affects the overall physical and psychological well-being of individuals living with the disease. The relationship between malnutrition and COPD negatively impacts the overall quality of life of patients, with common symptoms including increased fatigue, apathy, increased risk of falls, social isolation, and depression. The consequences of poor nutrition in this group indicate higher mortality rates [23].

## Nutritional Status Assessment in COPD

**Fig. 3:**
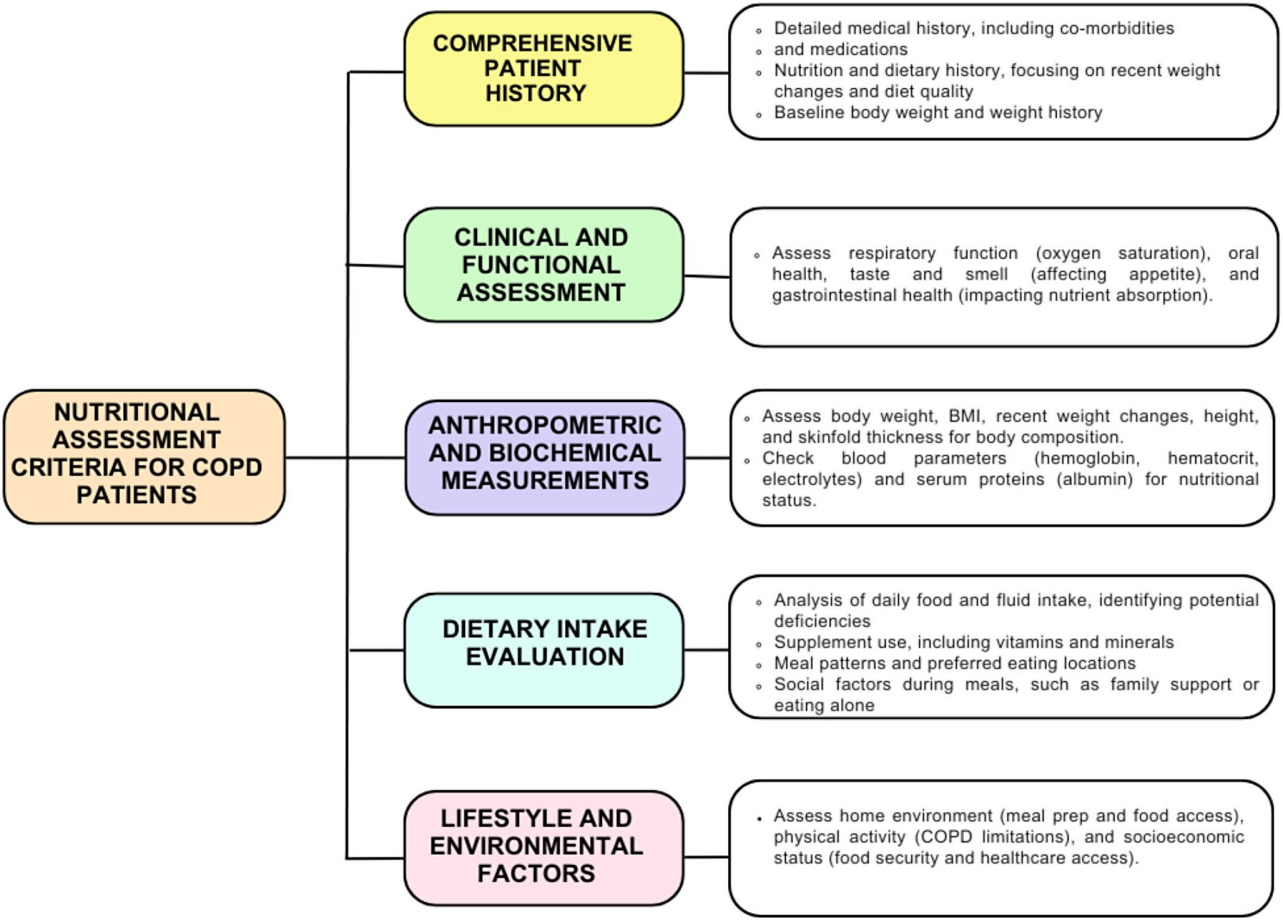
Nutritional assessment in COPD

The evaluation of nutritional status is gaining increasing importance in the management of COPD. Nutritional status is an essential indicator for both clinical outcomes and mortality [33]. Each COPD patient should be assessed individually. Factors such as age, gender, acute/chronic stages of the disease, other applied treatments, anthropometric measurements, bone mineral density, dietary intake, and activity level affect the nutritional therapy to be applied to the patient (Fig. 3) [34–36]. However, a single parameter does not fully reflect the overall nutritional status; therefore, a combination of various indicators should be used [37].

### Screening and Assessment Tools

**Fig. 4:**
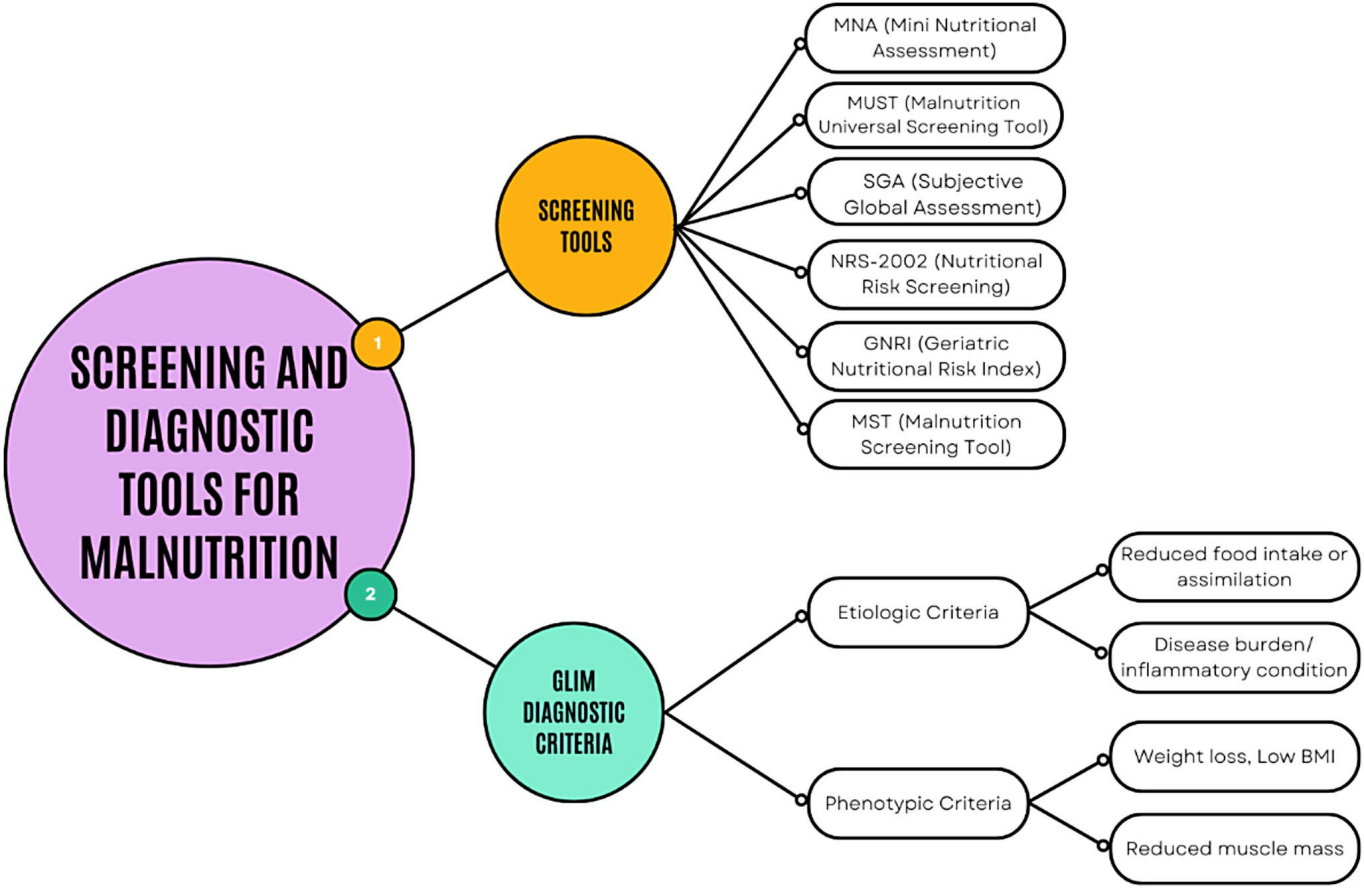
Screening and assessment tools in malnutrition

A nutritional assessment should be conducted in individuals at risk of malnutrition, and necessary steps such as nutritional therapy should be taken. Tools such as the Subjective Global Assessment (SGA) or the Mini Nutritional Assessment (MNA) can be used to identify individuals at risk of malnutrition (Fig. 4). These tools provide a quick screening based on clinical status, weight loss in recent months, nutrition over the last week, age, body weight, height, body mass index (BMI), body composition, and biochemical analyses [38, 39].

In contrast to screening tools, the Global Leadership Initiative on Malnutrition (GLIM) criteria, developed by an expert committee including global representatives from the European Society for Clinical Nutrition and Metabolism (ESPEN), the American Society for Parenteral and Enteral Nutrition (ASPEN), the Federación Latino Americana de Terapia Nutricional, Nutrición Clínica y Metabolismo (FELANPE), and the Parenteral and Enteral Nutrition Society of Asia (PENSA), offer a standardized diagnostic framework for confirming malnutrition [40, 41]. Introduced in 2018, GLIM involves a two-step process:

1. **Screening phase**: Tools such as MNA, MUST, or NRS-2002 are used to identify individuals at risk of malnutrition.
2. **Diagnostic phase**: Individuals identified as at risk undergo further evaluation using GLIM criteria, which include two etiologic (reduced food intake or assimilation, and disease burden/inflammatory condition) and three phenotypic (weight loss, low BMI, reduced muscle mass) indicators [42, 43].

These criteria are particularly valuable as they provide a globally standardized approach, bridging regional differences in malnutrition assessment. GLIM criteria address a significant gap by integrating both etiologic and phenotypic indicators into the diagnostic process, ensuring a more comprehensive evaluation. Given the particularly high risk of malnutrition in patients with Chronic Obstructive Pulmonary Disease (COPD), GLIM provides a robust framework for diagnosing malnutrition in this population, ensuring targeted and effective interventions [4].

The purpose of nutritional assessment is to determine risk based on the following results:

- Collect health history and conduct physical examination and biochemical analyses to identify any diseases or conditions that may cause nutritional deficiencies.
- Evaluate social and psychological inputs to determine the effects of living conditions, loneliness, and depression on nutrition.
- Obtain a dietary history, identifying barriers and needs in nutrition, including any conditions that limit food intake.
- Determine energy and fluid needs through indirect calorimetry or validated equations.
- Set protein needs between 0.8 g/kg/day (for healthy adults) and 1.5 g/kg/day (or higher in some cases) based on age, illness, and protein intake level.
- Determine micronutrient needs according to current recommendations and clinical status [44].

### Evaluation of Dietary Intake

Dietary history is a method that enables the estimation or assessment of a patient’s nutritional status. Through this method, information about the patient’s past and present eating habits, dietary intake, and food intolerances is collected. This information provides guidelines for the physician or clinician to prepare the most appropriate dietary prescription for each case.

A 24-hour dietary recall and food frequency can be used to assess COPD patients’ dietary intake. The 24-hour dietary recall method provides detailed information about current intake, including the number and timing of meals consumed the day before the interview. In some cases, dietary intake studies using the 24-hour dietary recall method are repeated over periods of 3, 5, 7, or more days.

Food frequency research presents a list of various foods and their consumption frequency [45]. Nutritional status in patients diagnosed with COPD is a poor prognostic factor. Additionally, other factors are thought to either protect against or contribute to disease progression. Adopting a healthy diet can help reduce the risk of COPD. A diet rich in antioxidants is associated with improved lung function and reduced long-term mortality in COPD patients. Therefore, maintaining an appropriate dietary pattern is crucial in COPD patients. Epidemiological evidence suggests that nutrition in COPD may be linked to the risk of disease progression [46, 47].

### Anthropometric Measurements

Anthropometry is a frequently used method for evaluating nutritional status due to its ease of application and low cost. Anthropometric measurements cover body dimensions and segments, such as height, weight, waist-to-hip ratio, BMI, length, and circumference measurements. BMI is a practical indicator among these measurements, though it does not reflect differences in body composition. BMI in COPD patients has been observed to have a significant impact on disease severity and quality of life. The Nutrition Screening Initiative has determined BMI values for COPD patients, suggesting that BMI can be a predictor of malnutrition or obesity [37, 48].

The BODE Index (Body mass index, airflow Obstruction, Dyspnea, and Exercise capacity) is a multidimensional scoring system designed to predict both the severity of respiratory and systemic components of COPD and the quality of life in these patients. It incorporates four variables: BMI (B), airflow obstruction (O) measured by the predicted FEV1%, dyspnea (D) assessed using the mMRC dyspnea scale, and exercise capacity (E) measured by the 6-minute walk test. In stable COPD patients, exercise-induced desaturation is a significant factor that can be used as a predictor of quality of life, future exacerbations, and disease severity. The BODE index provides valuable insights into disease progression and is a reliable tool for clinical decision-making in COPD management [49, 50].

Skinfold thickness measurement is a useful method for estimating body fat reserves, although it may be less accurate in elderly patients. Decreased muscle mass is an important indicator of malnutrition, and measurements of muscle circumference and arm muscle area can help assess this condition [37, 48].

***Evaluation of Muscle Mass and Body Composition Using Bioelectrical Impedance***.

The bio-impedance technique is used to measure an individual’s conductivity characteristics, thereby defining body composition and type, as well as determining fluid and tissue distribution. Estimation of body composition using bio-impedance is frequently used due to its ease of application and non-invasive nature. Emphysema and COPD patients typically have lower body fat percentages compared to individuals with chronic bronchitis and healthy subjects. Bio-impedance is a highly sensitive method for evaluating body composition in patients with chronic diseases. However, it shows low sensitivity in predicting changes in body composition within a short time frame. Literature also shows that bio-impedance is preferred over skinfold thickness measurement in assessing body composition in elderly COPD patients [37].

### Evaluation of Muscle Mass and Body Composition Using Computed Tomography (CT)

Various methods, such as bioelectrical impedance, dual X-ray absorptiometry, ultrasound, magnetic resonance imaging (MRI), and whole-body computed tomography (CT), are used to evaluate body composition. However, some of these methods have practical limitations in clinical settings, such as cost, time, and accessibility. Therefore, CT, particularly thoracic CT, is increasingly recognized in the COPD population given its clinical applications in characterizing parenchymal disease or screening for lung cancer. The use of CT in evaluating body composition has been described in various lung populations, including idiopathic pulmonary fibrosis, lung transplantation, and lung cancer [51]. Artificial intelligence algorithms are being developed to enable wider use of CT indices in clinical practice, providing significant potential for future research and clinical applications [33].

Most studies employ combined methods instead of a single method for nutritional assessment. In a study conducted by Ingadottir et al. with 121 COPD patients, the prevalence of malnutrition was assessed according to Icelandic Simple Screening (ISS), Nutritional Risk Screening (NRS-2002), and ESPEN criteria. Malnutrition was found at a rate of 36% with ISS, 55% with NRS-2002, and 21% with ESPEN criteria [17]. Another study by Marco et al. assessed malnutrition in 118 COPD patients using MNA-SF and ESPEN criteria. According to MNA-SF, 51.7% of patients were at risk of malnutrition, while ESPEN criteria identified malnutrition in 24.6%. These results indicate that MNA-SF identifies a broader group of patients at risk for malnutrition, whereas ESPEN criteria provide a more specific diagnostic ability [52]​​.

### Biochemical Findings

Biochemical parameters are important in assessing and monitoring the nutritional status of COPD patients. In malnourished patients, the creatinine-to-height index is used to evaluate lean body mass. Creatinine, a metabolite of creatine and phosphocreatine, is largely excreted by the body and is associated with the size of the muscle protein pool. Therefore, especially in COPD patients showing signs of muscle mass reduction, the creatinine-to-height index can be considered an important indicator for evaluating nutritional status [45].

## Nutritional Therapy in COPD

There are various mechanisms affecting respiratory function in COPD patients. Nutrition is a vital part of rehabilitation, particularly for individuals with chronic obstructive pulmonary disease [53]. The goal of nutritional therapy for patients at risk of nutrition-related complications due to exacerbations in COPD is to prevent malnutrition. For malnourished patients, the primary aim is to restore macro- and micronutrients to improve nutritional status.

Reversing malnutrition in COPD patients results in improved immune response by neutrophils and complements, thereby strengthening the body’s defense against infections. Improvements in respiratory muscle function, normalization of ventilatory response changes, and phosphatidylcholine synthesis rate in lung tissue and bronchoalveolar lavage fluid are observed, which normalizes surface tension forces. Despite being highly prevalent and a prognostic indicator of poor outcomes in COPD, malnutrition is recognized as a potentially modifiable independent risk factor with appropriate and effective dietary therapy [37].

### Goals of Nutritional Therapy

- To optimize the patient’s overall performance status and meet the demands of daily activities by preserving respiratory muscle strength, mass, and function.
- To maintain muscle mass and body fat reserves, consider changes in body composition.
- To correct fluid imbalances common in COPD patients.
- To manage drug-nutrient interactions that negatively affect food intake and nutrient absorption.
- To improve the patient’s quality of life [54].

Meeting the energy needs of these patients can be challenging. For those participating in pulmonary rehabilitation programs, energy requirements may increase or decrease depending on the intensity and frequency of exercise therapy. Since energy balance and nitrogen balance are interconnected, maintaining optimal energy balance is essential for preserving visceral and somatic proteins. Preferably, indirect calorimetry should be used to determine energy requirements and ensure sufficient but not excessive calorie provision. When energy equations are used to predict needs, adjustments for physiological stress should be included. Caloric needs can vary significantly from person to person and even within the same individual over time [55].

Omega-3 and omega-6 polyunsaturated fatty acids (PUFAs) are essential fats that cannot be synthesized by the body and must be obtained through the diet. The main components of omega-3, DHA, and EPA, may positively impact COPD treatment by reducing inflammation. PUFA supplementation is considered beneficial in COPD; however, its effectiveness depends on factors like duration of supplementation, adherence, and comorbidities. Additionally, DHA and AA supplementation has been shown to reduce the risk of upper respiratory infections and asthma. 1.2 to 1.5 g of protein per kilogram of dry body weight is necessary to maintain or regain lung and muscle strength and support immune function. A balanced ratio of protein (15–20% of calories), fat (30–45%), and carbohydrates (40–55%) is important to maintain a satisfactory respiratory quotient (RQ) from substrate metabolism. In patients with limited gas exchange ability, adequate but not excessive nutrition is crucial, as excessive calorie intake leads to CO₂ that must be expelled. Cardiovascular or renal disease, cancer, or diabetes also affect prescribed amounts, ratios, and types of protein, fat, and carbohydrates [56].

For patients with hypercapnia or those on ventilators, altering macronutrient composition is necessary. Consuming 4–6 small, nutrient-dense meals per day helps ease diaphragm movement and lung inflation. The consumption of fruits, vegetables, legumes, and whole grains rich in antioxidants, minerals, vitamins, flavonoids, phytochemicals, and fiber is recommended. Low serum vitamin D levels in COPD patients are significant due to their immunomodulatory effects and can help reduce muscle weakness. Vitamin C and E supplements also show promise in alleviating COPD symptoms. Limiting simple carbohydrates, especially table sugar, candies, pastries, and sodas, and favoring monounsaturated and polyunsaturated fats, typically in liquid form at room temperature and derived from plant sources, is advised. Foods that cause gas or bloating should be avoided, as they can make breathing more difficult. For COPD patients with chewing and swallowing difficulties, modified food textures (soft diet) are recommended. High fluid intake, generally 1 ml/kcal, is beneficial for thinning mucus secretions; however, limiting fluid intake during meals and consuming beverages an hour after meals is recommended [55].

Evidence suggests a clear link between certain dietary patterns and COPD progression. The Mediterranean diet, known for reducing the risk of respiratory diseases, stands out among effective dietary patterns. However, fast food consumption and Western eating habits are known to negatively impact COPD. Excessive consumption of red meat, processed meat, and sugary drinks, as well as reduced dairy intake, have been shown to worsen lung function. Conversely, a diet rich in whole grains, vegetables, fruits, and fish has been associated with a reduced risk of newly diagnosed COPD [57]. A diet rich in antioxidants is essential for maintaining health in COPD patients, as it is linked to improved lung function and reduced long-term mortality. Epidemiological evidence suggests that diet may be associated with the risk of disease progression in COPD [46, 58].

## Importance of Nutritional Supplements

In general, 10–45% of COPD patients are malnourished, and malnutrition is a significant prognostic factor for these patients [59]. Healthy and balanced nutrition should be encouraged in COPD patients. However, depending on the condition, nutritional supplements should be considered with a dietitian evaluation. Since COPD affects multiple organ systems, it emphasizes the need for multifaceted interventions, including various dietary supplements [54]. Numerous studies have examined the effects of nutritional supplements on COPD patients (Table 1).

A study conducted by Rafiq et al. investigated the effects of daily vitamin D supplementation on respiratory muscle strength and physical performance in 50 COPD patients with vitamin D deficiency. Participants were randomly assigned to receive either vitamin D supplementation (1200 IU daily) or a placebo. The study lasted for 6 months, with evaluations every 3 months. At the end of the study, serum 25(OH)D levels significantly increased in the group receiving vitamin D compared to the placebo group. However, there were no significant differences between the two groups in terms of respiratory muscle strength, physical performance measurements (maximum inspiratory and expiratory pressure, 6-minute walk test), pulmonary function, hand grip strength, exacerbation rate, and quality of life [60].

A study by Zendedel et al. aimed to evaluate the effects of vitamin D intake on FEV1 (forced expiratory volume in the first second) and COPD exacerbations in patients with severe and very severe COPD. The study was conducted in 2012 at Ashayer University Hospital, with 88 patients randomly assigned to either a vitamin D supplement group or a placebo group. In addition to routine COPD treatments, patients received either 100,000 IU of oral vitamin D or a placebo once a month for 6 months. Vitamin D intake reduced COPD exacerbations and improved FEV1 values in patients with severe and very severe COPD. These findings suggest that baseline serum vitamin D levels should be recorded in similar studies and vitamin D intake evaluated accordingly [61].​.

Deutz et al. evaluated the impact of a special nutritional supplement containing high protein and beta-hydroxy-beta-methylbutyrate (HP-HMB) on mortality risk compared to standard treatment in hospitalized elderly COPD patients with malnutrition. The study found that a specialized nutritional supplement could reduce mortality risk and improve overall health status in elderly COPD patients hospitalized with malnutrition. These findings suggest that nutritional supplements may play an important role in COPD management [62]. Another study evaluated the effects of essential amino acid (EAA) supplementation on physical performance and quality of life in severe COPD patients. The study included 88 COPD patients in GOLD class 3–4 (BMI < 23 kg/m²). EAA supplementation was found to help improve daily life performance, quality of life, nutritional and cognitive status, and muscle strength in severe COPD patients unable to participate in home rehabilitation programs [63]. Lu et al. evaluated the effects of oligomeric proanthocyanidins (OPCs) derived from grape seeds on antioxidant status and lung function in 27 COPD patients (13 in the intervention group and 14 in the placebo group). Patients received 150 mg of OPC or a placebo daily for 8 weeks. OPC supplementation increased antioxidant capacity and improved lipid profiles in COPD patients; however, there were no significant differences in lung function between the two groups [64].

The effects of omega-3 fatty acid (EPA + DHA) supplementation on protein metabolism, muscle mass, and muscle function in COPD patients were investigated. It was determined that both high-dose (3.5 g/day) and low-dose (2.0 g/day) EPA + DHA supplementation significantly increased muscle mass. Additionally, high-dose EPA + DHA supplementation was found to increase net protein synthesis by 15% and reduce postabsorptive protein breakdown by 13% [65]. The study by Engelen et al., 2024 investigated the effects of EPA/DHA (n-3 fatty acids) and HMB (β-hydroxy-β-methylbutyrate) supplementation in COPD patients. A total of 46 patients received either 2.0 g/day of EPA/DHA, 3.0 g/day of HMB + EPA/DHA, or a placebo for 10 weeks. The HMB + EPA/DHA group showed improvements in net protein breakdown (percentage increase) and the metabolism of glutamine, taurine, and tyrosine. Fat mass reduction and significant decreases in pro-inflammatory cytokines (IL-2, IL-17, IL-6, IL-12P40, TNF-β) were also observed. Both EPA/DHA and HMB + EPA/DHA supplementation increased lean soft tissue, while only EPA/DHA improved arginine, citrulline, and valine metabolism. The study highlighted the potential benefits of HMB in promoting fat mass loss and enhanced protein turnover in COPD patients [66]. Observational studies have suggested that omega-3 fatty acid intake, particularly EPA and DHA, may play a role in reducing respiratory symptoms and systemic inflammation in COPD patients. These findings complement interventional studies by highlighting the potential benefits of dietary omega-3 fatty acids in real-world populations [67, 68].

Paulin et al. conducted a study showing that vitamin B12 added to exercise training increased bicycle ergometer endurance in advanced COPD patients. The study was randomized and controlled, with patients receiving either vitamin B12 supplementation or a placebo for 8 weeks. Results showed significant improvements in exercise endurance and oxygen consumption in the vitamin B12 group. These findings suggest vitamin B12 may effectively increase exercise capacity in COPD patients [69].

Matheson et al. demonstrated that a specific oral nutritional supplement improved hand grip strength in hospitalized elderly patients with cardiovascular and pulmonary disease and malnutrition. In this randomized, double-blind controlled study, patients received either high-protein, beta-hydroxy-beta-methylbutyrate (HMB) containing oral nutritional supplements (ONS) or a placebo in addition to standard care. Results showed significant improvements in hand grip strength and nutritional status in the ONS group [70]. Additionally, the effect of magnesium was studied. Serum magnesium levels were investigated as a potential risk factor for acute COPD exacerbations in hospitalized elderly patients with cardiovascular and pulmonary disease and malnutrition. Findings suggest that low serum magnesium levels may increase the frequency of COPD exacerbations. Regular monitoring of magnesium levels and supplementation when necessary may be crucial in preventing COPD exacerbations [71].

For patients at risk of malnutrition or with a BMI < 20 kg/m², oral nutritional supplements are recommended to increase total calorie intake and promote weight gain. If no improvement is observed or further support is needed, a referral to a dietitian is advised. However, it is noted that malnutrition is still underrecognized in community and healthcare settings, where individuals are often inadequately nourished and hydrated. This may indicate that local guidelines do not reflect evidence-based guidance on malnutrition management, exposing patients to increased severity of malnutrition and poor clinical outcomes. Financial pressures on healthcare services mean that waiting times for therapeutic interventions can be long in some areas, which conflicts with guidelines recommending early intervention for at-risk patients. Early intervention is supported due to its significant cost benefits, as it allows earlier identification and treatment of patients at risk of malnutrition [32].

In a study investigating lung function, physical performance, and quality of life in patients with COPD in the stable phase with oral magnesium supplementation, 49 patients were divided into two groups receiving 300 mg/day magnesium citrate or placebo. At the six-month follow-up, a significant decrease in CRP levels, an indicator of inflammation, was observed in the magnesium group. Still, no significant difference was found in lung function, physical performance, and quality of life. These findings suggest that magnesium may have an anti-inflammatory effect but does not provide a significant improvement in COPD symptoms [72].

The effects of long-term high-dose vitamin C supplementation (2 g per day, 500 mg four times daily) were studied in COPD patients (*n* = 26). Participants were divided into two groups: those receiving standard COPD treatment and those receiving vitamin C in addition. After six months of follow-up, no significant difference in lung function was observed; however, there was a significant reduction in the exacerbation rate in the vitamin C group. These findings suggest that vitamin C may potentially be protective in COPD, but larger and longer-term studies are needed [73]. In the study examining the effects of Coenzyme Q10 and Creatine supplementation in COPD patients receiving long-term oxygen therapy, 90 of 108 patients participating in the study completed two months of treatment. In the group receiving supplementation, improvement was observed in the 6-minute walk test, body cell mass, phase angle, sodium/potassium ratio, dyspnoea indices and activities of daily living. Coenzyme Q10 plasma levels increased and differences in metabolomic profile were observed. These findings suggest that coenzyme Q10 and creatine may benefit COPD patients’ functional performance and body composition [74].

Gouzi et al. investigated the effect of antioxidant supplementation on pulmonary rehabilitation in COPD patients. 64 patients received antioxidant supplementation (alpha-tocopherol: 30 mg/day, ascorbate: 180 mg/day, zinc gluconate: 15 mg/day, selenomethionine: 50 µg/day) or placebo. Muscle strength (+ 11%), selenium levels (+ 16%), and serum total proteins (+ 7%) were significantly increased in the antioxidant group. The prevalence of muscle weakness decreased from 30 to 10.7%. However, there was no additional improvement in muscular endurance. Supplements may benefit muscle strength and other secondary parameters during pulmonary rehabilitation [75].

In a meta-analysis by Huang and Ko, which included 29 randomized controlled trials with 1625 participants (mean age 67.9 years), nutritional supplements significantly improved body weight, lean mass index, and the 6-minute walk test. However, no significant effects were found on hand grip or quadriceps muscle strength [76]. Dietary micronutrient supplementation appears to have important clinical effects for COPD patients.

**Table 1:** Characteristics and main outcomes of nutritional interventions and dietary intake studies in COPD patients

Author(s) and Year	Study Type	ONS/Nutrient Supplement or Factor Assessed	Participants (*N*)	Treatment Duration	Intervention/Evaluation Method	Main Results
*Rafiq et al.*, *2017*	Randomized Controlled Trial	Vitamin D	50 COPD patients (24 in vitamin D group, 26 placebo)	6 months	1200 IU vitamin D daily or placebo	Increase in serum 25(OH)D level; no differences in respiratory muscle strength, physical performance, pulmonary function, exacerbation rate, or QoL.
*Zendedel et al.*,* 2015*	Randomized Controlled Trial	Vitamin D	88 COPD patients (44 in vitamin D group, 44 placebo)	6 months	Monthly 100,000 IU vitamin D or placebo	Improvement in FEV1 values and reduction in exacerbation frequency.
*Deutz et al.*,* 2021*	Randomized Controlled Trial	HP-HMB	214 COPD patients (109 in HP-HMB group, 105 placebo)	3 months	HP-HMB enriched nutritional supplements or placebo	Reduced mortality risk, improved handgrip strength, weight gain, and nutritional biomarkers (serum hemoglobin, calcium, 25-hydroxy vitamin D).
*Dal Negro et al.*,* 2012*	Randomized Controlled Trial	Essential Amino Acids (EAA)	88 COPD patients (44 in EAA group, 44 placebo)	12 weeks	2 doses per day (4 g) of essential amino acids or placebo	Improvements in physical activity, significant enhancement in St. George Respiratory Questionnaire score, increased serum albumin, muscle strength.
*Lu et al.*,* 2018*	Randomized Controlled Trial	Proanthocyanidins (OPCs)	27 COPD patients (13 in OPC group, 14 placebo)	8 weeks	150 mg OPC daily or placebo	Increase in HDL-C levels and reduction in oxidative stress markers; no significant difference in lung function.
*Engelen et al.*,* 2022*	Randomized Controlled Trial	EPA + DHA (n-3 PUFA)	32 COPD patients (12 high dose, 10 low dose, 10 placebo)	4 weeks	High EPA + DHA (3.5 g) or low EPA + DHA (2 g) dose, and placebo	High-dose EPA + DHA: Increase in net protein synthesis and reduction in postabsorptive protein breakdown. Both groups: Increase in muscle mass.
*Engelen et al.*,* 2024*	Randomized Controlled Trial	HMB + EPA/DHA (n-3 PUFA)	46 COPD patients (16 EPA/DHA, 14 HMB + EPA/DHA, 16 placebo)	10 weeks	Daily 2.0 g EPA/DHA or 3.0 g HMB + EPA/DHA or placebo	HMB + EPA/DHA: Reduction in net protein breakdown, decrease in fat mass, and proinflammatory cytokines. EPA/DHA: Increase in lean soft tissue mass.
*Kemper et al.*,*2024*	Prospective Observational Study	Plasma Omega-3 (EPA + DHA)	57 former smokers with moderate-to-severe COPD	6 months	Plasma omega-3 levels measured via gas chromatography/mass spectrometry; assessed quality of life and exacerbation frequency using SGRQ and clinical metrics.	Higher plasma EPA + DHA levels associated with better respiratory-specific quality of life (lower SGRQ score by 2.7 points per SD increase) and reduced odds of moderate exacerbations (by 60%).
Table 1**(continued).** Characteristics and main outcomes of nutritional interventions and dietary intake studies in COPD patients						
**Author(s) and Year**	**Study Type**	**ONS/Nutrient Supplement or Factor Assessed**	**Participants (N)**	**Treatment Duration**	**Intervention/Evaluation Method**	**Main Results**
*Lemoine et al.*,*2019*	Cross-Sectional Study	Omega-3 (ALA, EPA, DHA)	878 U.S. adults with COPD	Not applicable (cross-sectional study)	Dietary intake assessed using 24-hour dietary recalls; respiratory symptoms evaluated through NHANES questionnaire.	Higher ALA intake associated with reduced odds of respiratory symptoms (chronic cough and wheeze) at lower omega-6 intake. EPA + DHA intake showed benefits only in subgroups of current smokers with low socioeconomic status.
*Paulin et al.*,* 2017*	Randomized Controlled Trial	Vitamin B12	32 COPD patients (8 rehab + B12, 8 rehab, 8 B12 only, 8 placebo)	8 weeks	Daily 500 mcg vitamin B12 or placebo	Significant improvement in exercise tolerance; no major changes in oxygen consumption kinetics. B12 deficiency observed in 34.4% of advanced COPD.
*Matheson et al.*,* 2020*	Randomized Controlled Trial	ONS (High Protein + HMB)	652 patients (328 ONS, 324 placebo)	90 days (hospital and post-discharge)	2 portions of ONS daily or placebo	14.1% increase in handgrip strength (HGS), improvement in nutritional status, and association with daily activity improvement.
*Kshirsagar et al.*,* 2021*	Observational Study	Serum Magnesium (Mg²⁺)	100 COPD patients (72 hypomagnesemic, 28 normomagnesemic)	1-year follow-up	Serum magnesium levels (< 1.7 mg/dL)	72% hypomagnesemia prevalence. Serum Mg²⁺ <1.7 mg/dL patients had 9.34 times higher exacerbation risk. Increased severity was linked to hypomagnesemia.
*Zanforlini et al.*,* 2022*	Randomized Controlled Trial	Magnesium Citrate	49 COPD patients (25 intervention, 24 placebo)	6 months	Daily 300 mg magnesium citrate or placebo	Reduction in CRP levels in the intervention group at 6 months. No significant difference in lung function, physical performance, or quality of life. Potential anti-inflammatory effects.
*Gouzi et al.*,* 2019*	Randomized Controlled Trial	Vitamin C, Vitamin E, Zinc, Selenium	64 COPD patients (32 antioxidant group, 32 placebo)	28 days	Daily 180 mg Vitamin C, 30 mg Vitamin E, 15 mg Zinc, 50 µg Selenium, or placebo	11% increase in muscle strength, reduction in muscle weakness, no difference in muscle endurance.
*Dey et al.*,* 2021*	Randomized Controlled Trial	Vitamin C	26 COPD patients (14 vitamin C group, 12 placebo)	6 months	Daily 2 g Vitamin C or standard treatment	Reduction in exacerbation frequency, improvement in quality of life, no significant difference in respiratory function.
*De Benedetto et al.*,* 2018*	Randomized Controlled Trial	Coenzyme Q10 and Creatine	90 COPD patients (45 Coenzyme Q10 group, 45 placebo group)	2 months	Twice daily 160 mg Coenzyme Q10 and 170 mg Creatine, or placebo	Increased 6-minute walking test distance, reduction in inflammatory metabolites, and improvement in muscle function.

## Conclusion

Chronic Obstructive Pulmonary Disease (COPD) is a widespread health issue globally, significantly impacting patients’ quality of life. Studies have shown that malnutrition is a common and serious problem among COPD patients, which may worsen the disease course, increase symptoms, and reduce treatment response.

Nutrition is a critical component in COPD treatment. Especially in managing COPD, nutritional status assessment is becoming increasingly important, as it is a significant indicator for both clinical outcomes and mortality. Therefore, the goal of nutritional therapy is to preserve respiratory muscle strength, mass, and function, optimize overall patient performance status, preserve muscle mass and body fat reserves due to changes in body composition, correct fluid imbalances, control drug-nutrient interactions that negatively affect food intake and nutrient absorption, and improve the patient’s quality of life.

Nutritional supplementation may be initiated if deemed appropriate after nutritional assessment. Supplements such as vitamin D, antioxidants, and omega-3 fatty acids may slow disease progression and improve general health. However, due to conflicting study results, more clinical studies are needed in this area to observe the long-term effects of nutritional interventions. Additionally, nutritional assessments and personalized dietary therapies are crucial in COPD management and should be integrated to improve patients’ prognosis. These findings highlight the role of nutrition in COPD treatment and recommend greater attention to nutritional interventions in clinical practice.

In summary, monitoring nutritional status and implementing appropriate nutritional interventions in COPD patients is vital to prevent disease progression and improve patients’ quality of life. As part of a holistic approach to COPD management, personalized nutritional therapies should be integrated, and the role of nutrition should be further emphasized in clinical applications.

## Key References

**Nguyen**, **H.T.**,** et al. (2020)**. *Effectiveness of Tailored Dietary Counseling in Treating Malnourished Outpatients with Chronic Obstructive Pulmonary Disease: A Randomized Controlled Trial*. Journal of the Academy of Nutrition and Dietetics, 120(5), 778–791.e1. 10.1016/j.jand.2020.01.009.

- This study evaluates tailored dietary counseling’s efficacy in managing malnutrition in COPD outpatients. Its findings underscore the importance of individualized nutritional interventions, directly supporting the personalized approach emphasized in this manuscript.

**Engelen**,** M.P.K.J.**,** et al. (2024)**. *Functional and Metabolic Effects of Omega-3 Polyunsaturated Fatty Acid Supplementation and the Role of β-Hydroxy-β-Methylbutyrate Addition in Chronic Obstructive Pulmonary Disease: A Randomized Clinical Trial*. Clinical Nutrition, 43(9), 2263–2278. 10.1016/j.clnu.2023.06.012.

- This clinical trial reveals omega-3 and HMB supplementation’s metabolic benefits in COPD patients. It highlights nutritional strategies to enhance functional outcomes, complementing the manuscript’s discussion on therapeutic supplementation.

**Beijers**,** R.J.**,** et al. (2023)**. *The Role of Diet and Nutrition in the Management of COPD*. European Respiratory Review, 32(168). 10.1183/16000617.0172-2023.

- This review explores the interplay between nutrition and COPD management, offering a comprehensive perspective on dietary interventions for disease mitigation, reinforcing the manuscript’s focus on the role of nutrition in chronic conditions.

## Data Availability

No datasets were generated or analysed during the current study.

## References

[1] Gayan-Ramirez G. Relevance of nutritional support and early rehabilitation in hospitalized patients with COPD. J Thorac Dis. 2018;10(Suppl 12):S1400–14. 29928522 10.21037/jtd.2018.03.167 PMC5989102

[2] GOLD. Global Initiative for Chronic Obstructive Lung Disease (GOLD) guidelines for chronic obstructive pulmonary disease. Global Initiative for Chronic Obstructive Lung Disease (GOLD); 2024.

[3] Deng M, et al. Global prevalence of malnutrition in patients with chronic obstructive pulmonary disease: systemic review and meta-analysis. Clin Nutr. 2023;42(6):848–58. 37084471 10.1016/j.clnu.2023.04.005

[4] Kaluźniak-Szymanowska A et al. Optimal Assessment of Nutritional Status in older subjects with the Chronic Obstructive Pulmonary Disease-A comparison of three screening tools used in the GLIM Diagnostic Algorithm. Int J Environ Res Public Health, 2022. 19(3). 10.3390/ijerph19031025 PMC8834573 35162048

[5] Nguyen HT, et al. Effectiveness of tailored Dietary Counseling in Treating Malnourished outpatients with Chronic Obstructive Pulmonary Disease: a Randomized Controlled Trial. J Acad Nutr Dietetics. 2020;120(5):778–e7911. 10.1016/j.jand.2019.09.013 31786177

[6] Nguyen L, et al. Nutrition Therapy in End-Stage Lung Disease. Curr Nutr Rep. 2017;6(3):291–8.

[7] Kahnert K, et al. The diagnosis and treatment of COPD and its comorbidities. Dtsch Arztebl Int. 2023;120(25):434–44. 36794439 10.3238/arztebl.m2023.027 PMC10478768

[8] Keogh E, Williams EM. Managing malnutrition in COPD: a review. Respir Med. 2021;176:106248. 33253970 10.1016/j.rmed.2020.106248

[9] MacLeod M, et al. Chronic obstructive pulmonary disease exacerbation fundamentals: diagnosis, treatment, prevention and disease impact. Respirology. 2021;26(6):532–51. 33893708 10.1111/resp.14041

[10] PRASAD B. Chronic obstructive pulmonary disease (COPD). Int J Pharm Res Technol (IJPRT). 2020;10(1):67–71.

[11] Heefner A, et al. The role of Nutrition in the Development and Management of Chronic Obstructive Pulmonary Disease. Nutrients. 2024;16(8):1136. 38674827 10.3390/nu16081136 PMC11053888

[12] Mete B et al. Prevalence of malnutrition in COPD and its relationship with the parameters related to disease severity. Int J Chronic Obstr Pulm Dis, 2018: pp. 3307–12. 10.2147/COPD.S179609 PMC6188194 30349235

[13] Beijers RJ, Steiner MC, Schols AM. The role of diet and nutrition in the management of COPD. Eur Respiratory Rev, 2023. 32(168). 10.1183/16000617.0003-2023 PMC10245132 37286221

[14] Akner G, Larsson K. Undernutrition state in patients with chronic obstructive pulmonary disease. A critical appraisal on diagnostics and treatment. Respir Med. 2016;117:81–91. 27492517 10.1016/j.rmed.2016.05.023

[15] Kahnert K, et al. The diagnosis and treatment of COPD and its comorbidities. Deutsches Ärzteblatt International. 2023;120(25):434. 36794439 10.3238/arztebl.m2023.027 PMC10478768

[16] Hancu A. Nutritional status as a risk factor in COPD. Maedica (Bucur). 2019;14(2):140–3. 31523295 10.26574/maedica.2019.14.2.140 PMC6709393

[17] Ingadottir AR, et al. Two components of the new ESPEN diagnostic criteria for malnutrition are independent predictors of lung function in hospitalized patients with chronic obstructive pulmonary disease (COPD). Clin Nutr. 2018;37(4):1323–31. 28641831 10.1016/j.clnu.2017.05.031

[18] BAPEN, *Managing Malnutrition in COPD*. 2020.

[19] Mancin S, et al. Prevention and Management of Malnutrition in patients with Chronic Obstructive Pulmonary Disease: a scoping review. Adv Respir Med. 2024;92(5):356–69. 39311113 10.3390/arm92050034 PMC11417808

[20] Roberts MH, et al. Disease Burden and Health-Related Quality of Life (HRQoL) of Chronic Obstructive Pulmonary Disease (COPD) in the US - evidence from the Medical Expenditure Panel Survey (MEPS) from 2016–2019. Int J Chron Obstruct Pulmon Dis. 2024;19:1033–46. 38765766 10.2147/COPD.S446696 PMC11100519

[21] Igboekwe E, Verma S, Paczkowski R. Real-world disease burden and healthcare resource utilization among patients with COPD and asthma using triple therapy (FF/UMEC/VI) in the United States. Int J Chronic Obstr Pulm Dis, 2024: pp. 281–96. 10.2147/COPD.S423993 PMC10824610 38292138

[22] Collins PF et al. Nutritional support in chronic obstructive pulmonary disease (COPD): an evidence update. J Thorac Disease, 2019: pp. S2230–7. 10.21037/jtd.2019.10.41 PMC6831917 31737350

[23] Wilson N, Turner S. Targeting malnutrition in patients with COPD in the community. Br J Nurs. 2023;32(21):S6–12. 38006589 10.12968/bjon.2023.32.21.S6

[24] Ma K, et al. Pathogenesis of Sarcopenia in chronic obstructive pulmonary disease. Front Physiol. 2022;13:850964. 35928562 10.3389/fphys.2022.850964 PMC9343800

[25] Kim SH, et al. Sarcopenia Associated with Chronic Obstructive Pulmonary Disease. J Bone Metab. 2019;26(2):65–74. 31223602 10.11005/jbm.2019.26.2.65 PMC6561852

[26] Tramontano A, Palange P. Nutritional State and COPD: effects on Dyspnoea and Exercise Tolerance. Nutrients. 2023;15(7):1786. 37049625 10.3390/nu15071786 PMC10096658

[27] De Brandt J, et al. Update on the etiology, Assessment, and management of COPD Cachexia: considerations for the Clinician. Int J Chron Obstruct Pulmon Dis. 2022;17:2957–76. 36425061 10.2147/COPD.S334228 PMC9680681

[28] Rôlo Silvestre C, et al. The Nutritional Status of Chronic Obstructive Pulmonary Disease Exacerbators. Can Respir J. 2022;2022:3101486. 36276928 10.1155/2022/3101486 PMC9584732

[29] de Blasio F, et al. Malnutrition and sarcopenia assessment in patients with chronic obstructive pulmonary disease according to international diagnostic criteria, and evaluation of raw BIA variables. Respir Med. 2018;134:1–5. 29413494 10.1016/j.rmed.2017.11.006

[30] Rawal G, Yadav S. Nutrition in chronic obstructive pulmonary disease: a review. J Transl Int Med. 2015;3(4):151–4. 27847905 10.1515/jtim-2015-0021 PMC4936454

[31] Jiang R, et al. Causal relationship between chronic obstructive pulmonary disease and BMD at different sites: a bidirectional mendelian randomization study. Medicine. 2023;102(41):e35495. 37832103 10.1097/MD.0000000000035495 PMC10578729

[32] Shepherd AB, Bowell K. *‘Mind the gap’: the importance of managing malnutrition in chronic obstructive pulmonary disease.* British Journal of Nursing, 2019. 28(22): pp. 1442–1449. 10.12968/bjon.2019.28.22.1442 31835938

[33] Beijers R, Steiner MC, Schols A. The role of diet and nutrition in the management of COPD. Eur Respir Rev, 2023. 32(168). 10.1183/16000617.0003-2023 PMC10245132 37286221

[34] Schols AM, et al. Nutritional assessment and therapy in COPD: a European Respiratory Society statement. Eur Respir J. 2014;44(6):1504–20. 25234804 10.1183/09031936.00070914

[35] Rawal G, Yadav S. Nutrition in chronic obstructive pulmonary disease: a review. J Translational Intern Med. 2015;3(4):151–4. 10.1515/jtim-2015-0021 PMC4936454 27847905

[36] Yang P-H, et al. Effect of nutritional intervention programs on nutritional status and readmission rate in malnourished older adults with pneumonia: a randomized control trial. Int J Environ Res Public Health. 2019;16(23):4758. 31783672 10.3390/ijerph16234758 PMC6926802

[37] Alibakhshi E, Shirvani H. Nutritional status in patients with chronic obstruction pulmonary disease (COPD)-Review article. EC Nutr. 2015;2(1):267–74.

[38] Serón-Arbeloa C et al. Malnutrition Screening and Assessment. Nutrients, 2022. 14(12). 10.3390/nu14122392 PMC9228435 35745121

[39] Cortés-Aguilar R, et al. Validity of nutrition screening tools for risk of malnutrition among hospitalized adult patients: a systematic review and meta-analysis. Clin Nutr. 2024;43(5):1094–116. 38582013 10.1016/j.clnu.2024.03.008

[40] Wang Y, et al. Evaluation of different screening tools as the first step of the GLIM framework: a cross-sectional study of Chinese cancer patients in an outpatient setting. Nutr Clin Pract. 2024;39(3):702–13. 38161144 10.1002/ncp.11103

[41] Cederholm T, et al. GLIM criteria for the diagnosis of malnutrition - A consensus report from the global clinical nutrition community. Clin Nutr. 2019;38(1):1–9. 30181091 10.1016/j.clnu.2018.08.002

[42] Henriksen C, et al. Agreement between GLIM and PG-SGA for diagnosis of malnutrition depends on the screening tool used in GLIM. Clin Nutr. 2022;41(2):329–36. 34999327 10.1016/j.clnu.2021.12.024

[43] Jazinaki MS, et al. Two-step GLIM approach using NRS-2002 screening tool vs direct GLIM criteria application in hospital malnutrition diagnosis: a cross-sectional study. Nutr Clin Pract. 2024;39(6):1419–30. 39446911 10.1002/ncp.11229

[44] Kılıç L. *Vücut Kompozisyonunun Değerlendirilmesi-Nutrisyonel Destek.* 2019.

[45] Fernandes AC, Bezerra OMdPA. Nutrition therapy for chronic obstructive pulmonary disease and related nutritional complications. Jornal Brasileiro De Pneumologia. 2006;32:461–71. 17268751

[46] Mekal D, Czerw A, Deptala A. Dietary Behaviour and Nutrition in patients with COPD treated with long-term oxygen therapy. Int J Environ Res Public Health. 2021;18(23):12793. 34886519 10.3390/ijerph182312793 PMC8657430

[47] Nguyen HT, et al. Effectiveness of tailored dietary counseling in treating malnourished outpatients with chronic obstructive pulmonary disease: a randomized controlled trial. J Acad Nutr Dietetics. 2020;120(5):778–91. e1. 10.1016/j.jand.2019.09.013 31786177

[48] Srigiripura CV, et al. Determinants of malnutrition and associated parameters in subjects with stable chronic obstructive pulmonary disease: a cross sectional study. North Afr J Food Nutr Res. 2023;7(16):85–100.

[49] Pandu V, et al. Evaluation of correlation of Oxygen Desaturation with Bode Index in stable COPD patients. Int J Health Sci. 2022;6(S6):3184–92.

[50] Li CL, et al. Using the BODE Index and comorbidities to predict health utilization resources in Chronic Obstructive Pulmonary Disease. Int J Chron Obstruct Pulmon Dis. 2020;15:389–95. 32110007 10.2147/COPD.S234363 PMC7036670

[51] Nicholson JM, et al. Computed tomography-based body composition measures in COPD and their association with clinical outcomes: a systematic review. Chron Respir Dis. 2022;19:14799731221133387. 36223552 10.1177/14799731221133387 PMC9561670

[52] Marco E, et al. Malnutrition according to ESPEN consensus predicts hospitalizations and long-term mortality in rehabilitation patients with stable chronic obstructive pulmonary disease. Clin Nutr. 2019;38(5):2180–6. 30342931 10.1016/j.clnu.2018.09.014

[53] Rondanelli M, et al. Food pyramid for subjects with Chronic Obstructive Pulmonary diseases. Int J Chron Obstruct Pulmon Dis. 2020;15:1435–48. 32606652 10.2147/COPD.S240561 PMC7310971

[54] Collins P, Weekes CE. *Respiratory disease.* Manual of dietetic practice (5th Edition), 2014: pp. 375–384.

[55] Elfagi S, Nouh F, Omar M. Chronic obstructive Pulmonary diseases Nutritional Guideline. EAS J Nutr Food Sci. 2020;2:55–61.

[56] Raymond JL, Morrow K. Krause and mahan’s food and the nutrition care process e-book. Elsevier Health Sciences; 2020.

[57] Rondanelli M et al. Food pyramid for subjects with chronic obstructive pulmonary diseases. Int J Chronic Obstr Pulm Dis, 2020: pp. 1435–48. 10.2147/COPD.S240561 PMC7310971 32606652

[58] Scoditti E, et al. Role of Diet in Chronic Obstructive Pulmonary Disease Prevention and Treatment. Nutrients. 2019;11(6):1357. 31208151 10.3390/nu11061357 PMC6627281

[59] Hsieh MJ, Yang TM, Tsai YH. Nutritional supplementation in patients with chronic obstructive pulmonary disease. J Formos Med Assoc. 2016;115(8):595–601. 26822811 10.1016/j.jfma.2015.10.008

[60] Rafiq R et al. Effects of daily vitamin D supplementation on respiratory muscle strength and physical performance in vitamin D-deficient COPD patients: a pilot trial. Int J Chronic Obstr Pulm Dis, 2017: pp. 2583–92. 10.2147/COPD.S132117 PMC5584776 28894361

[61] Zendedel A, et al. Effects of vitamin D intake on FEV1 and COPD exacerbation: a Randomized Clinical Trial Study. Glob J Health Sci. 2015;7(4):243–8. 25946929 10.5539/gjhs.v7n4p243 PMC4802087

[62] Deutz NE, et al. Reduced mortality risk in malnourished hospitalized older adult patients with COPD treated with a specialized oral nutritional supplement: sub-group analysis of the NOURISH study. Clin Nutr. 2021;40(3):1388–95. 32921503 10.1016/j.clnu.2020.08.031

[63] Dal Negro R, et al. Essential amino acid supplementation in patients with severe COPD: a step towards home rehabilitation. Volume 77. Monaldi archives for chest disease; 2012. 2. 10.4081/monaldi.2012.154 23193843

[64] Lu M-C, et al. Effect of oligomeric proanthocyanidin on the antioxidant status and lung function of patients with chronic obstructive pulmonary disease. vivo. 2018;32(4):753–8. 10.21873/invivo.112304 PMC6117753 29936455

[65] Engelen MPKJ, et al. ω-3 polyunsaturated fatty acid supplementation improves postabsorptive and prandial protein metabolism in patients with chronic obstructive pulmonary disease: a randomized clinical trial. Am J Clin Nutr. 2022;116(3):686–98. 35849009 10.1093/ajcn/nqac138 PMC9437982

[66] Engelen MPKJ, et al. Functional and metabolic effects of omega-3 polyunsaturated fatty acid supplementation and the role of β-hydroxy-β-methylbutyrate addition in chronic obstructive pulmonary disease: a randomized clinical trial. Clin Nutr. 2024;43(9):2263–78. 39181037 10.1016/j.clnu.2024.08.004

[67] Kemper TA, et al. Higher plasma omega-3 levels are associated with improved exacerbation risk and respiratory-specific quality of life in COPD. Chronic Obstr Pulmonary Diseases: J COPD Foundation. 2024;11(3):293. 10.15326/jcopdf.2023.0468 PMC11216231 38687147

[68] Lemoine S. Omega-3 fatty acid intake and prevalent respiratory symptoms among US adults with COPD. BMC Pulm Med. 2019;19:1–9. 31122230 10.1186/s12890-019-0852-4 PMC6533751

[69] Paulin FV, et al. Addition of vitamin B12 to exercise training improves cycle ergometer endurance in advanced COPD patients: a randomized and controlled study. Respir Med. 2017;122:23–9. 27993287 10.1016/j.rmed.2016.11.015

[70] Matheson EM, et al. Specialized oral nutritional supplement (ONS) improves handgrip strength in hospitalized, malnourished older patients with cardiovascular and pulmonary disease: a randomized clinical trial. Clin Nutr. 2021;40(3):844–9. 32943241 10.1016/j.clnu.2020.08.035

[71] Kshirsagar K, Patil VC. Chronic obstructive pulmonary disease: is serum magnesium level a risk factor for its acute exacerbation? Casp J Intern Med. 2021;12(2):223–7. 10.22088/cjim.12.2.223 PMC8111813 34012542

[72] Zanforlini BM, et al. Clinical trial on the effects of oral magnesium supplementation in stable-phase COPD patients. Aging Clin Exp Res. 2022;34(1):167–74. 34260036 10.1007/s40520-021-01921-z PMC8794984

[73] Dey D, Sengupta S, Bhattacharyya P. Long-term use of Vitamin-C in chronic obstructive pulmonary disease: early pilot observation. Lung India, 2021. 38(5). 10.4103/lungindia.lungindia_959_20 PMC8509159 34472536

[74] De Benedetto F, et al. Supplementation with Qter^®^ and creatine improves functional performance in COPD patients on long term oxygen therapy. Respir Med. 2018;142:86–93. 30170808 10.1016/j.rmed.2018.08.002

[75] Gouzi F, et al. Additional effects of Nutritional antioxidant supplementation on peripheral muscle during Pulmonary Rehabilitation in COPD patients: a Randomized Controlled Trial. Volume 2019. Oxidative Medicine and Cellular Longevity; 2019. p. 5496346. 1. 10.1155/2019/5496346 PMC6501222 31178967

[76] Huang W-J, Ko C-Y. Systematic review and meta-analysis of nutrient supplements for treating Sarcopenia in people with chronic obstructive pulmonary disease. Aging Clin Exp Res. 2024;36(1):69. 38483650 10.1007/s40520-024-02722-w PMC10940388

